# Circulating Cytokines and Growth Factors in Pediatric Pulmonary Hypertension

**DOI:** 10.1155/2012/143428

**Published:** 2012-12-18

**Authors:** Mark Duncan, Brandie D. Wagner, Keri Murray, Jenna Allen, Kelley Colvin, Frank J. Accurso, D. Dunbar Ivy

**Affiliations:** ^1^Section of Pediatric Pulmonology, School of Medicine, University of Colorado and Children's Hospital Colorado, Aurora, CO 80045, USA; ^2^Obesity Research Center, King Saud University, Riyadh, Saudi Arabia; ^3^Biodesix Inc., Boulder, CO 80045, USA; ^4^Division of Endocrinology, Metabolism and Diabetes, School of Medicine, University of Colorado Denver, MS 8106, 12801 E. 17th Avenue, RC 1 South, Room 7103, Aurora, CO 80045, USA; ^5^Department of Biostatistics and Informatics, Colorado School of Public Health, University of Colorado Denver, Aurora, CO 80045, USA; ^6^Section of Pediatric Cardiology, School of Medicine, University of Colorado and Children's Hospital Colorado, Aurora, Colorado 80045, USA

## Abstract

*Background*. Management of pediatric pulmonary hypertension (PH) remains challenging. We have assessed a panel of circulating proteins in children with PH to investigate their value as predictive and/or prognostic biomarkers. From these determinations, we aim to develop a practical, noninvasive tool to aid in the management of pediatric PH. *Methods*. Twelve cytokines and growth factors putatively associated with lung or vascular disease were examined in plasma specimens from 70 children with PH using multiplex protein array technology. Associations between hemodynamics, adverse events, and protein markers were evaluated. *Results*. Epidermal growth factor (EGF) and IL-6 were associated with important hemodynamics. Of the twelve proteins, VEGF and IL-6 were significantly, univariately associated with the occurrence of an adverse event, with odds ratios (95% confidence intervals) of 0.56 (0.33–0.98) and 1.69 (1.03–2.77), respectively. When hemodynamic predictors were combined with protein markers, the ability to predict adverse outcomes within the following year significantly increased. *Conclusions*. Specific circulating proteins are associated with hemodynamic variables in pediatric PH. If confirmed in additional cohorts, measurement of these proteins could aid patient care and design of clinical trials by identifying patients at risk for adverse events. These findings also further support a role for inflammation in pediatric PH.

## 1. Introduction

Pulmonary hypertension (PH) encompasses a diverse group of disorders, all characterized by an elevation in pulmonary arterial pressure and pulmonary vascular resistance (PVR) [1]. Pediatric PH is associated with significant morbidity and mortality, and differs from PH as manifest in adults in several important ways, such as the development of PH in a growing lung [1]. Medical treatment of pediatric PH has been challenging due to a lack of accessible, noninvasive, objective measures that can aid in managing these patients [2]. Specifically, there is a need for better tools for prognosis and assessment of the progression of the disease [1, 3, 4]. 

Outcomes of most interest to clinicians and PH patients include death, hospitalization for PH-related events, right ventricular failure, initiation of intravenous prostanoid therapy, and lung transplantation. Although definitive, these end points are often difficult to study in pediatric PH due to their infrequent occurrences and the requirement for long-term followup. Currently, disease progression is most commonly assessed through a 6-minute walk distance and hemodynamic assessments, but both of these suffer from practical drawbacks and neither is thoroughly validated in the pediatric population. These limitations in current outcomes and prognostic variables have spurred our search for reliable, noninvasive, objective, and cost-effective alternatives that are easily measured in children [1, 4]. Circulating protein biomarkers have the potential to meet these objectives [5].

The role of inflammation in the pathobiology of PH has recently been emphasized [6–9]. Cytokine- and growth factor-dependent mechanisms lead to inflammatory cell recruitment and are prominent in PH [9–11]. Of these factors, interleukin (IL)-6 is possibly the most studied [7, 12–15]. For example, IL-6 administration in rats induces PH, and IL-6 augments hypoxia-induced PH. In addition, it has been reported recently that transgenic mice overexpressing IL-6 display PH, vascular remodeling, and an exaggerated response to hypoxia [16]. Further, circulating levels of cytokines are reportedly important in the pathogenesis of a wide range of other conditions including chronic heart failure [17], acute renal failure [18], and sepsis [19].

Recent developments in protein array technology allow high throughput, multiplex analysis with excellent sensitivity, precision, and specificity. The approach is also cost effective and uses minimal sample because all the analytes are measured on a single chip simultaneously. The requirement for low sample volumes is of special interest in the management of pediatric patients. We therefore applied a protein array approach that provides accurate and precise quantification of twelve proteins with known lung or vascular effects in a cohort of pediatric patients with PH. Our objective was to determine whether there is a clinically relevant association between any of the panel constituents, individually or combined, with hemodynamic parameters, disease prognosis, and relevant adverse outcomes. Our hypothesis was that measurement of these factors could be prognostic in pediatric PH. 

## 2. Materials and Methods

### 2.1. Patient Population

Prior approval for these studies was obtained from the Colorado Multiple Institutional Review Board (COMIRB, approval #05-0551). Written informed consent was obtained from the parents or guardians of all children who served as subjects of the investigation, and when appropriate, from the subjects themselves. These studies comply with the Health Insurance Portability & Accountability Act of 1996. Plasma samples were collected from 70 pediatric patients with PH ranging in age from newborn to 21.3 years old. Samples were obtained between November 2001 and May 2008. All patients were being treated with appropriate therapies to manage their PH during sample collection (Table 1) [20]. Patients were classified as idiopathic pediatric pulmonary arterial hypertension (IPAH) or associated pulmonary arterial hypertension (APAH) according to published criteria [2]. 

### 2.2. Data Variables

In this study, disease prognosis/severity was determined using hemodynamic parameters measured in the cardiac catheterization laboratory during a routine pulmonary hypertension visit. The data were obtained with the patient breathing room air, prior to testing pulmonary reactivity with the use of vasodilator therapy. For right-heart catheterization, we used a Swan-Ganz catheter in the femoral vein or internal jugular vein to determine mean right atrial pressure (RAP), mean pulmonary artery pressure (PAP), pulmonary capillary wedge pressure (PCWP), cardiac index (CI), and pulmonary vascular resistance index (PVRi). Invasive arterial monitoring was used to measure the systemic vascular resistance index (SVRi). We used Fick with assumed oxygen consumption in those patients with shunts and thermodilution in all others to measure cardiac output and then calculate cardiac index. Most subjects were ventilated because they were children. In addition, the reactivity to inhaled nitric oxide was calculated by taking the difference in mean PAP between room air and with nitric oxide. The adverse outcome used in the predictive models was defined as initiation of intravenous prostanoids (epoprostenol or treprostinil), transplantation, and/or death occurring within 12 months from the collection of the blood sample. Admission for right heart failure is a rare event in children and was not included in the adverse outcome definition. 

### 2.3. Circulating Protein Measurements

Blood (5 mL) was drawn, plasma isolated, and this was frozen at −70°C until analysis. Protein determinations were performed on an Evidence Analyzer (Randox Laboratories, Northern Ireland) according to the manufacturer's protocol for the simultaneous quantification of the following analytes: interleukin-1alpha (IL-1*α*), interleukin-1beta (IL-1*β*), interleukin-2 (IL-2), interleukin-4 (IL-4), interleukin-6 (IL-6), interleukin-8 (IL-8), interleukin-10 (IL-10), tumor necrosis factor alpha (TNF*α*), gamma interferon cytokine (IFN*γ*), monocyte chemotactic protein-1 (MCP-1), endothelial growth factor (EGF), and vascular endothelial growth factor (VEGF). Values for individual proteins measured by this multiplexed protein array technology have been shown to correlate with single ELISA measurements. Further, intra- and interassay coefficients of variation of less than 10% are typical for most protein measured by this multiplexed approach with this system [21]. Determinations were made in duplicate and all calculations were based on the average of the two measurements for each sample. Values below the limit of detection were set to a value of 0. 

### 2.4. Statistical Analysis

Descriptive statistics were calculated using mean ± standard deviations or medians and the interquartile range (IQR) for continuous variables and percentages for categorical variables. Protein measurements were transformed (log base 2) and anchored at 1. Comparisons across groups were made by Wilcoxon rank sum or chi-squared tests, as appropriate. Spearman rank correlation coefficients were used to estimate the association between the hemodynamic variables and protein markers. Logistic regressions were fit to investigate the association between the dichotomous adverse outcome variable (outcome observed within 12 months) and the protein measurements. Mean standardization and principal component analysis (PCA) were performed for dimension reduction across the protein measurements, resulting in a single value representing a weighted combination of all twelve markers. A score statistic was used to select the principal component (PC) most associated with the outcome and the best subset of two PCs and clinical variables in a multivariate logistic regression. The predictive ability of the model was investigated using a *c*-index (area under the ROC curve). The improvement in risk prediction associated with the addition of the protein biomarker PC was assessed by calculation of a net reclassification improvement (NRI) measure [22]. To estimate the predictive ability of the logistic regressions to an independent set of observations, *c*-indices were calculated using the leave-out-one cross-validation approach. All analyses were performed using SAS version 9.2 software (SAS Institute Inc., Cary, NC, USA, 2008).

## 3. Results

**Figure 1:**
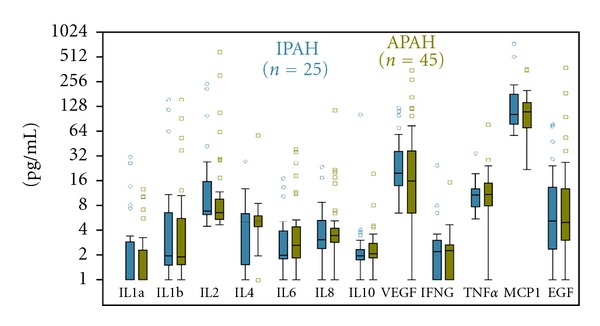
Biomarker characteristics: the distribution of the 12 cytokine and growth factor measurements based on disease classification. The *y*-axis is displayed on the log (base 2) scale. Filled areas indicate the interquartile range (distance between 25th and 75th percentiles), the middle line corresponds to the median, and the whiskers contain data within 1.5 interquartile ranges. None of the differences was statistically significant.

The study population consisted of 70 pediatric patients with PH: 36% IPAH and 64% with APAH. Of these, 80% of patients were treated with at least one therapy (Table 1). The median PAP was elevated at 34 mm Hg (the interquartile range (IQR) was 23–56 mm Hg) with a median RAP value of 5 mm Hg (IQR, 3–7 mm Hg). Nine (13%) patients experienced an adverse event within 12 months of sample collection. These patients had worse mPAP levels ranging from 30 to 76 with a median of 65.5 mm Hg as well as a median PVRI of 15.5 Wood Units × m^2^ ranging from 5.1–20.4. Only 39 of the 48 patients with vasoreactivity tests had complete data in order to assess the responder status based on the pediatric definition [23]. Based on these data 2 (5%) were classified as responders and neither of these patients experienced an adverse event. Individual protein measurements are displayed separately by diagnostic group (Figure 1). There were no significant differences between the IPAH and APAH groups for any of the protein levels. 

Cardiac catheterization was performed for all patients in the initial evaluation for PH. Thereafter, only 59 (84%) of the patients had a catheterization performed on the same day as blood collection for the correlation analysis, and of these, 48 individuals also had a vasoreactivity test in response to inhaled nitric oxide (NO). The following pairs of hemodynamic and circulating protein markers had correlation coefficients (*r*) significantly different from zero: IL-1*α* and CI (*r* = −0.31, CL = −0.53 to −0.04); EGF and mPAP (*r* = −0.45, CL = −0.63 to −0.21); EGF and PVRi (*r* = −0.37, CL = −0.57 to −0.12); EGF and PVR/SVR (*r* = −0.42, CL = −0.62 to −0.17); IL-6 and mPAP (*r* = 0.27, CL = 0.01 to 0.49); IL-6 and PVRi (*r* = 0.32, CL = 0.06 to 0.53). None of the biomarkers correlated with the change in mean PAP in response to inhaled NO. 

**Figure 2:**
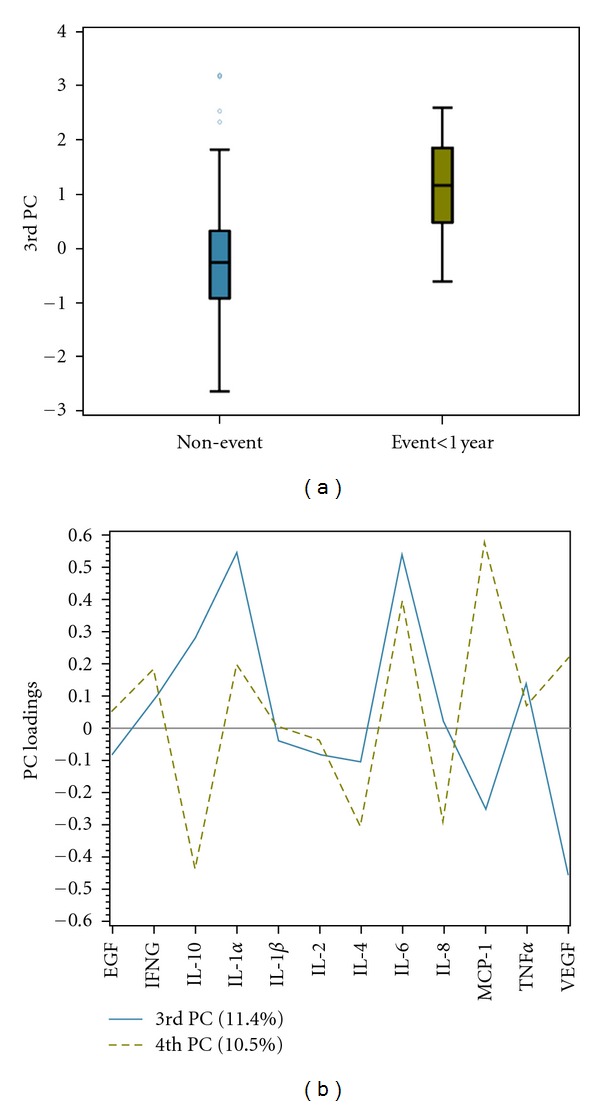
Dimension reduction using principal component analysis: the third PC was found to be the most associated with outcome. (a) The distribution of the 3rd PC is displayed separately by outcome. Filled areas indicate the interquartile range (distance between 25th and 75th percentiles), the middle line corresponds to the median, and the whiskers contain data within 1.5 interquartile ranges. (b) The PC loadings for the 3rd and 4th PCs are displayed. The 3rd PC is heavily loaded by positive IL-1*α* and IL-6 and negative VEGF values, whereas the 4th PC is heavily weighted by positive IL-6 and MCP-1 and negative IL-10 values.

To assess the potential additive predictive ability of the proteins we measured, a series of logistic regression models predicting adverse outcome within 1 year were estimated. The first of these were univariate models that were estimated to determine the association of each protein individually with the adverse outcome variable. VEGF and IL-6 were significantly associated with the adverse outcome: (OR (95% CI) were as follows: 0.56 (0.33–0.98) and 1.69 (1.03–2.77), respectively. In addition to VEGF, IL-1*β*, IL-2, IL-4, MCP1, and EGF also had negative associations, indicating that lower values of these proteins were associated with the adverse event outcome. PCA was performed on the protein marker data to condense the 12 measurements to a few orthogonal components. The 3rd component was significantly associated with the outcome, with higher values corresponding to an increased risk of an adverse event. The risk associated with this protein index was weighted by higher values of IL-1*α* and IL-6 and lower VEGF values (Figure 2). This finding corresponds well with the results from the univariate analyses and gave a *c*-index of 0.81.

**Figure 3:**
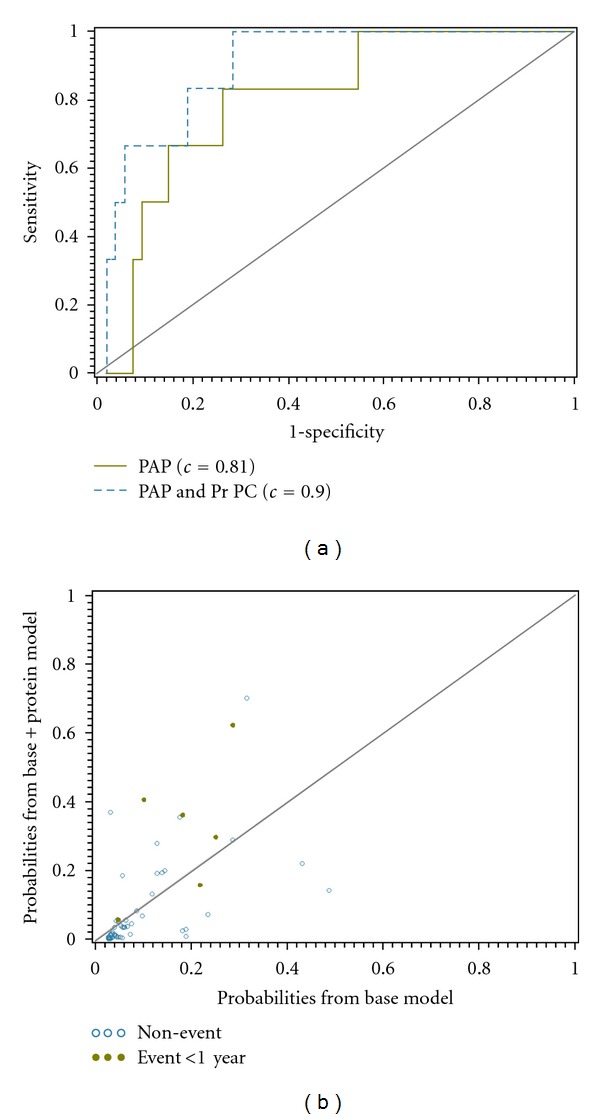
Added value of proteins toward outcome prediction. (a) Comparison of the ROC curves indicate an improvement in discriminative ability with the addition of the 4th PC calculated from the protein measurements. *c*-indices for each model are also displayed. (b) The reclassification of estimated probabilities for the logistic regression model which included protein markers as a predictor versus the model based on PAP alone. The dots represent those observations associated with an event and the open circles with a nonevent. Higher percentages of events and nonevents are desired above and below the reference line, respectively. The corresponding reclassification index was significant (*P* = 0.01) indicating a significant improvement in prediction with the addition of the proteins.

Score statistics were used to identify the top predictive clinical markers and determine the added value of a protein marker index over the top clinical predictor. The resulting clinical model identified PAP as the top predictor. Using this model as a base model, the PCs of the protein measurements were added as predictors and the selection process was repeated. After mPAP, the 4th PC was the top predictor indicating it contains information orthogonal to the hemodynamic variable. The risk associated with the 4th PC was weighted by higher values of MCP1 and IL-6 and lower IL-10 values. To evaluate the added predictive value of the protein measurements in addition to mPAP, ROC curves, corresponding *c*-indices, and NRI measures were utilized (Figure 3). The inclusion of these two proteins increased the *c*-index from 0.81 to 0.90. The predicted probabilities from both models were also compared. Significant improvement was seen with the addition of the protein measurements (NRI *P* value = 0.01) indicating that their inclusion enhanced the models ability to correctly classify patients. This result was verified with a resampling approach used to estimate the models predictive ability when applied to an independent set of observations. The estimated *c*-indices for the model with mPAP alone, and with mPAP and the protein measurements, were 0.69 and 0.82, respectively.

## 4. Discussion 

Our data (Table 1) indicate a surprisingly high morbidity and mortality, even in children with mild pulmonary hypertension, and they underscore the fact that this is a very high-risk group. This observation stimulated our efforts to develop a practical and noninvasive approach to the management of pediatric pulmonary hypertension that can be used in a routine setting. Of interest and clinical relevance is the fact that we have shown that by simply adding the quantification of a set of plasma proteins, it is possible to markedly improve our ability to predict outcomes in children with PH. Specifically, the addition of a protein index, weighted mostly by IL-6, IL-10, and MCP-1 concentrations, significantly increased the probabilities for those patients with an adverse event compared to hemodynamic measurements alone. When investigating the plasma proteins univariately, VEGF and IL-6 were associated with occurrence of an adverse event and EGF and IL-6 were correlated with mean PAP and PVRi. In addition, we identified a combination of all twelve proteins which associated well with adverse events and had similar predictive ability compared to the top hemodynamic predictor. 

Interest in the role of cytokines and growth factors in the development of PH has grown. Recent evidence is consistent with the view that circulating factors and inflammatory cells contribute to remodeling in chronic PH [11, 24, 25]. Circulating and lung levels of the proinflammatory cytokine IL-6 are increased in patients with idiopathic PH and PH associated with inflammatory diseases [12–15]. IL-6 may also be a mediator of disease in PH because overexpression of IL-6 in a mouse model exacerbated PH by both proproliferative and antiapoptotic mechanisms [16]. Other studies confirm an important role for IL-6 in PH. For example, IL-6 treated mice are prone to develop hypoxia-induced pulmonary hypertension [26] which may be mediated in part by an inflammatory process [27]. In our study, we found that increased levels of circulating IL-6 were significantly associated with an increased risk of an adverse event but when considered multivariately with PAP, CI, and VEGF, it was replaced with an anti-inflammatory cytokine, IL-10. Interestingly, IL-6 and IL-10 levels were positively associated with each other. 

Recently there has also been increased interest in the role of VEGF in pulmonary arterial hypertension. Studies indicate VEGF may be an important mediator of lung growth. VEGF is responsible for angiogenesis and vasculogenesis, and animal models indicate that when VEGF signaling is impaired, this contributes to the pathogenesis of PH [28, 29]. Alternatively, growth factors may be important for the maintenance of continued lung growth in patients with pulmonary arterial hypertension, and a favorable phenotype may be associated with increased VEGF and EGF. Farkas et al. [30] found a similar inverse relationship between VEGF and PAP in rats and Lassus et al. [31] reported decreased circulating plasma levels of VEGF in human infants with PH. 

The search for biomarkers that are useful in the management of pediatric PH is in its infancy, but the ideal marker would be obtained noninvasively, easily measured, and would offer high sensitivity and specificity. Currently, the brain natriuretic peptide system and neurohumoral markers have been evaluated in children with PH [32–34]. 

Our study demonstrates that quantification of a panel of cytokines and growth factors has potential applications in clinical trial design by identifying patients at risk for an adverse event. In addition, patients at higher risk for an adverse event may need to be treated more aggressively with continuous intravenous therapy or listed for lung transplantation.

## 5. Conclusions

This study adds to the growing body of literature indicating that inflammation is important in pediatric PH and that circulating (blood) biomarkers can be important tools in prediction and prognostic evaluation of pediatric patients with PH. It is envisioned that future use of biomarkers will guide appropriate treatment selection. The potential for growth factors, EGF and VEGF, to aid in the management of PH is important and novel. Serial sampling and assessment of longitudinal changes might provide important additional insights, but these findings indicate that even a single point in time determination can predict the future clinical course. We intentionally examined a heterogeneous group of patients in this study in order to explore patterns of biomarker expression. It is clear that the next step is to validate these findings in a larger scale study including several groups of well-characterized subjects. We suggest that, based on our findings, additional evaluation of cytokines and growth factors is warranted because there is growing evidence that these determinations may be relevant for disease management. 

## Figures and Tables

**Table 1:** Patient demographics and clinical measurements.

Patient demographics (no of subjects = 70)	
Female—no. (%)	37 (52.9%)
APAH—no. (%)	45 (64.3%)
Congential heart disease	31 (44.3%)
Chronic lung disease	11 (15.7%)
Clotting disorders	10 (14.3%)
Age (years)—median (IQR)	8.0 (4.4–13.0)
Therapies	
Mono therapy	23 (32.9%)
Dual therapy	23 (32.9%)
Triple therapy	10 (14.3%)
Calcium channel blockers	22 (31.4%)
PDE-5 inhibitors	29 (41.4%)
Endothelin receptor blockers	25 (35.7%)
Prostacyclin	23 (32.9%)
Epoprostenol	12 (17.1%)
IV Treprostinil	8 (11.4%)
Inh Iloprost	3 (4.3%)
CATH variables—median (IQR) (*n* = 59)	
Pulmonary artery pressure, mm Hg	34 (23–56)
Pulmonary capillary wedge pressure, mm Hg	8 (6–10)
Right atrial pressure, mm Hg	5 (3–7)
Cardiac index, L/min × m^2^	3.5 (3.0–4.3)
Pulmonary vascular resistance index, wood units × m^2^	5.6 (4.1–13.9)
PVR/SVR	0.48 (0.27–0.76)
Vasoreactivity (% change in mPAP w/NO)	−21.1 (−29.5 to −12.8)
Follow-up time (months)—median (min–max)	36 (12–89)
Adverse outcomes	16 (22.9%)
Within 12 months	9 (12.9%)
First observed outcome	
Expired	10
Transplantation	0
Initiation of IV prostanoids	8

## Abbreviation

APAH: Associated pulmonary arterial hypertension
CI: Cardiac index
EGF: Endothelial growth factor
IFN*γ*: Gamma interferon cytokine
IL: Interleukin
IPAH: Idiopathic pulmonary arterial hypertension
IQR: Interquartile range
NO: Nitric oxide
MCP-1: Monocyte chemoattractant protein-1
NRI: Net reclassification improvement
PAP: Pulmonary artery pressure
PCWP: Pulmonary capillary wedge pressure
PH: Pulmonary hypertension
PVR: Pulmonary vascular resistance
RAP: Right arterial pressure
SVR: Systemic vascular resistance
TNF*α*: Tumor necrosis factor alpha
VEGF: Vascular endothelial growth factor.
